# ICECAP-O, the current state of play: a systematic review of studies reporting the psychometric properties and use of the instrument over the decade since its publication

**DOI:** 10.1007/s11136-019-02114-y

**Published:** 2019-01-21

**Authors:** Louise Proud, Carol McLoughlin, Philip Kinghorn

**Affiliations:** Health Economics Unit, Institute of Applied Health Research, University of Birmingham, B15 2TT Edgbaston, UK

**Keywords:** ICECAP-O, Capability approach, Systematic review, Economic evaluation, Validity, Responsiveness

## Abstract

**Purpose:**

A paper reporting the development of the ICECAP-O was published in 2006. Since then, there has been increasing interest in the use of capability-based measures within health economics and the ICECAP-O has been suggested for use in economic evaluation by decision-making bodies in the Netherlands and UK.

**Methods:**

A systematic review of studies published between January 2006 and October 2018 which have assessed the psychometric properties of ICECAP-O or utilised the measure within economic evaluation.

**Results:**

Twenty-four studies explored the psychometric properties of ICECAP-O and 21 have utilised the measure within economic evaluation; one study reported psychometric properties as well as utilising the measure within economic evaluation. The ICECAP-O has good construct validity and responsiveness, but there is evidence of some issues relating to content validity. In the context of economic evaluation, the ICECAP-O has, to date, mainly been included as a secondary economic measure and the reporting of results is brief with minimal detail and often no discussion. Five of the economic evaluation studies combined scores from ICECAP-O with time, but each used different terminology to describe this result.

**Conclusion:**

Focus, in terms of publications, appears to have shifted now from assessment of psychometric properties to the utilisation of the ICECAP-O within economic evaluation. Further research is needed with respect to a decision-rule for the ICECAP measures. This additional research should also guide users in terms of appropriate analysis, terminology and presentation of results, which are in-keeping with the conceptual framework underpinning the ICECAP-O.

**Electronic supplementary material:**

The online version of this article (10.1007/s11136-019-02114-y) contains supplementary material, which is available to authorized users.

## Background

There has been growing interest in recent years in operationalising Amartya Sen’s capability approach in the context of health economics [1]. One of the first capability-based measures of well-being to be developed and used within health economics was the ICECAP-O [2], a measure of well-being for older adults (aged 65 years and older) with five attributes, each with four response levels. The attributes are: Attachment (love and friendship); Security (thinking about the future without concern); Role (doing things that make you feel valued); Enjoyment (enjoyment and pleasure); Control (independence). The paper outlining the development of ICECAP-O was published in 2006 [2] and since then three other capability measures have been developed: The ICECAP-A (for the general adult population) [3], the ICECAP-SCM (for use in the context of supportive end of life care) [4], and the ICECAP-CPM (to assess the well-being of ‘close persons’ in the context of end of life care) [5].

The ICECAP measures have each been developed to assess well-being and are therefore relevant for use in contexts where a focus on health functioning alone is likely to present a partial or misleading picture of the benefits of an intervention. As an example, the Zorginstituut in the Netherlands recommends the inclusion of ICECAP alongside EQ-5D for the evaluation of interventions in long-term care, where the relevant outcomes extend beyond health [6]. In 2013, the National Institute for Health and Care Excellence (NICE) in the UK extended its remit to include social care and when publishing guidance on the methods for economic evaluation of social care, NICE suggest the use of capability measures, including ICECAP [7].

Tariff values have been elicited for ICECAP-O from a sample of older people in the UK using best-worst scaling [8]. Values are anchored on a scale of zero (no capability) to one (full capability), and hence, the ICECAP-O cannot be used to calculate quality-adjusted life years, where the scaling is that of zero (dead) to one (full health). Valuation reflects an intentional differentiation from cost-utility analysis, with a conceptual alignment instead to the Capability Approach of Sen and Nussbaum [1, 2, 8].

As the ICECAP-O has now been in the public domain for over a decade, we present a systematic review of studies which have either assessed the psychometric properties of the measure, or have used the measure to collect data.

## Methods

A systematic search of the literature was undertaken to identify studies which had assessed the psychometric properties of the ICECAP-O or reported use of the measure in economic evaluation. Methods were based on the UK Centre for Review and Dissemination (CRD) guidelines [9] and the Cochrane Handbook for Systematic Reviews [10]; results are reported in line with the Preferred Reporting Items for Systematic Reviews and Meta-Analyses (PRISMA) guidelines [11].

### Scoping search and preliminary research

A background scoping search was undertaken in May 2017, which included searches conducted using study names from the ICECAP-O study database.^1^ The list of papers identified in this way was used to inform the development and refinement of the definitive search terms and strings by checking whether or not search terms detected these key papers. The scoping search suggested that only a limited number of studies have been undertaken to date, indicating that using deliberately broad search terms would maximise the number of studies captured (high sensitivity). However, using the term ‘ICECAP’ alone would not be feasible due to the large number of irrelevant papers identified. The scoping search also revealed that many papers that report having used ICECAP-O do not mention the measure in the title, abstract or keywords.

### Identification of relevant studies

The search strategy (informed by the earlier scoping search) aimed to identify all studies that have assessed the psychometric properties of ICECAP-O or used the measure to collect data for inclusion within economic evaluation. It was designed to be as inclusive as possible whilst also being feasible. A search of electronic databases was undertaken in October 2018 and involved using the following predetermined keywords:

- ICECAP-O
- ICEPOP
- Investigating Choice Experiments

Seven databases were searched: SCOPUS, PUBMED CENTRAL, ProQuest Science and Technology, EMBASE, CINAHL Plus, Nursing and Allied Health Database and ProQuest Social Sciences Premium Collection. The search terms were combined using the Boolean logic term ‘OR’ to keep the search broad.

The first paper reporting the development of the ICECAP-O was published in 2006 [2]; therefore, the search results were limited to material from January 2006 to October 2018. No restrictions were imposed in relation to study participants, interventions, study design or setting, and both published and unpublished materials were included. Given the scoping search revealed that many studies which have used ICECAP-O do not make reference to this in the title or abstract, the search was expanded to cover full texts where the database allowed for this.

Identified papers were then compared with those located in the scoping search, to identify any papers not captured by the electronic database search. Manual searching of the reference lists of papers selected for review was also undertaken, to identify any additional relevant studies. The search strategy was reviewed by a medical librarian.

### Study selection

The process for study selection comprised of the following two stages (screening and then assessment of eligibility).

First, records were excluded as not relevant if they were:

- published as a conference *abstract* only (no full text available);
- not published in English;
- Provided a commentary only or reported a study design other than an assessment of psychometric properties or an economic evaluation;
- a full-text paper that did not contain at least one of the search terms of interest, in the title, abstract or main body of the paper.

Remaining papers were read in full and assessed against the inclusion criteria. Papers were included if they reported a study that had done at least one of the following:

- assessed the psychometric properties of the ICECAP-O;
- used the ICECAP-O to measure outcomes for economic evaluation (including pilot and feasibility trials);

The search revealed that sometimes the same study was reported in more than one paper. The unit of interest in this review is studies rather than papers; therefore, papers sharing the same author, trial registration number, study name or study settings were identified and cross-referenced to link together multiple reports of the same study.

### Data extraction

Two data extraction forms (Supplementary Table 1) were developed; one for studies assessing psychometric properties of ICECAP-O and one for studies using the measure to collect data.

### Analysis plan

Given the anticipated diversity of studies (in terms of objective; the country within which data was collected; the population group; and intervention (where relevant)), a narrative synthesis was used [9]. Studies were initially categorised as those:

- Investigating psychometric properties of ICECAP-O
- Reporting use of the ICECAP-O in economic evaluation

Following initial, textual description of the two categories of studies, those in the first category were further grouped using headings and terminology from the COSMIN taxonomy [12]:

- Validity (further defined as: criterion, content and construct)
- Reliability (commonly test–retest)
- Responsiveness (defined as the ability of a measure to detect clinically important changes resulting from an intervention [13])

Results from studies reporting on validity, reliability and responsiveness were summarised in tabular form, with textual discussion of the relationship between studies. Evidence of a relationship was determined by the size of the correlation *p* value at the following levels [14]:

- *p* > 0.1: no significant evidence of a relationship
- 0.1 ≥ *p* > 0.01: weak evidence of a relationship
- *p* ≤ 0.01: strong evidence of a relationship

## Results

### Search results and study selection

The scoping search identified 26 published papers meeting the inclusion criteria.

**Fig. 1:**
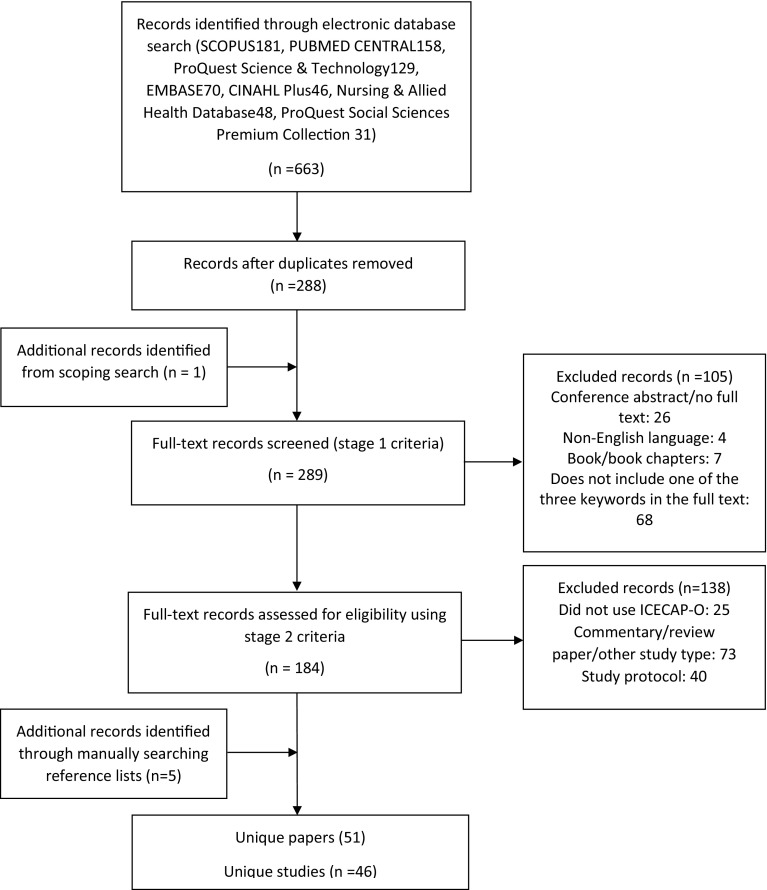
Flow diagram of search results and study selection

The full search generated 288 unique records, including 25 of the 26 papers identified through the scoping search. The paper identified through the scoping search but not through the full electronic database search was added prior to screening (stage one), to make a total of 289 papers. One hundred and five papers were excluded at the first stage. A further 138 papers were excluded at stage two. Five additional papers were identified through a search of the reference lists of papers included after stage two. There were a total of 51 full-text papers, relating to 46 unique studies. Figure 1 provides a flow diagram of the records identified and included or excluded at each stage. Twenty-four studies solely reported an assessment of the psychometric properties of the measure. Twenty-one studies solely reported use of the ICECAP-O in full or partial economic evaluations (including pilot and feasibility studies). One paper [15] both assessed psychometric properties and reported data from a feasibility study. All but two studies were reported in published papers: the reference by Keeley [16] is a doctoral thesis, and the reference by Flynn et al. [17] a working paper.

**Fig. 2:**
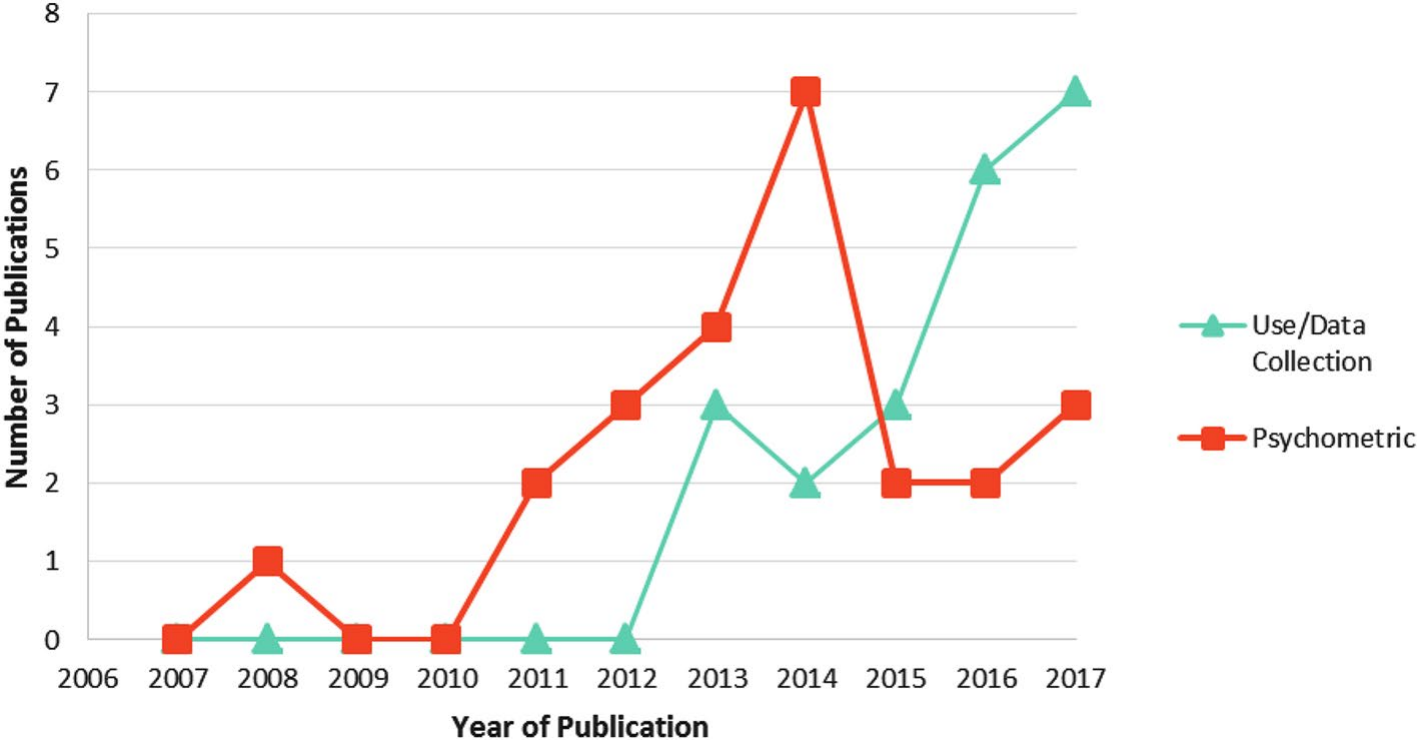
Publications assessing the psychometric properties of or reporting use of ICECAP-O

Figure 2 plots the number of unique publications per year, categorised as either assessing the psychometric properties of ICECAP-O or reporting the use of the measure for data collection. It can be seen that the number of publications assessing psychometric properties peaked at seven in 2014, and that there was then a lag before a peak in the number of papers reporting use of the measure (seven papers in 2017). Data from 2018 is excluded as the search was conducted part way through that year, as is the paper by Milne [15] which could have been added in both categories.

### Studies assessing the psychometric properties of ICECAP-O

#### Overview of studies

All but eight [15, 18–24] of the 25 studies identified as reporting psychometric properties of ICECAP-O (including the paper by Milne et al. [15]) assessed construct validity. (Specific methodology referred to by authors was convergent, divergent and discriminant validity.) Responsiveness and reliability have received much less attention: five studies assessed responsiveness [16, 22, 24–26], just two assessed reliability [23, 26]. Content validity (including face, item and sampling validity) was assessed in six studies [15, 18–21, 23]. The majority of studies (21) in this category were quantitative studies. The remaining four used qualitative or semi-qualitative techniques and comprised: two ‘think aloud’ studies [19, 21], one study based on semi-structured interviews [20], and one semi-qualitative-quantitative study based on the Nominal Group Technique [18].

The psychometric properties of the ICECAP-O were assessed across a range of patient and general populations: five focussed on the general population only [23, 27–30]; Couzner et al. included both patients (with post-acute needs) and the general population [31]. Of those studies which exclusively included patients/service users, five focused on cognitive impairment [15, 32–35], three on frailty/social care needs [21, 26, 36] and two on those at high risk of falling [22, 37, 38]. Two studies focussed on patients with post-acute needs [39–41]. One study included patients who had previously had a stroke [16] and one study included the carers of people with dementia [20]. Five studies focussed on patients undergoing joint surgery [17–19, 24, 42].

The majority of studies were undertaken in Europe with ten conducted in the UK [15–20, 24, 27, 29, 36], five in the Netherlands [21, 26, 30, 34, 39], and one each in Germany [33], Spain [35] and Sweden [23]. Of the remaining studies, four were carried out in Australia [28, 31, 40–42] and three in Canada [22, 32, 37, 38].

Supplementary Table 2 provides a full summary of papers reporting psychometric properties.

#### Construct Validity

A summary of results relating to the assessment of construct validity is presented in Table 1. Of those studies that examined construct validity, the comparators most commonly used can be broadly categorised as ‘sociodemographic characteristics’, ‘general health’, ‘Activities of Daily Living/physical independence’, ‘mental health (non-dementia specific)’, ‘cognitive-impairment’ ‘well-being’ and ‘environment and care quality’. Each is examined in turn.

**Table 1:** Results relating to construct validity

Comparator	Evidence (statistical significance) of relationship with overall capability [statements in square brackets indicate the degree to which the results were in line with the hypothesis]
Socio-demographic characteristics	
Increased age	None [expected] [40, 43] Negative [NH] [42] ICECAP-O does not discriminate between over and under 65’s [NH] [28] ICECAP-O discriminates between the young-old (65–75) and old-old (over 75) [expected] [29, 33, 35, 36, 39]
Living with others	None [positive expected] [28] Positive [expected] [29] None [NH] [42]
Living with marital partner	None [positive expected] [28]
Generic health measures (index score)	
EQ-5D-3L or 5L	Positive strong [expected] [36] Positive [expected] [16, 38, 44] Positive [expected] [34] Positive [expected] [39] Positive [expected] [33] Positive [NH] [42] ICECAP-O discriminates between: above and below average health [NH] [36] patients and the general population [expected] [45]
Physical health/independence	
Katz Index of Independence in Activities of Daily Living (ADL)	ICECAP-O discriminates between IADL dependent and non-IADL dependent elderly [expected] [39]
Barthel activities of daily living (ADL) Index	Positive strong [expected] [33, 36] Positive strong [expected] [35]
Mental health (non-dementia)	
Geriatric depression scale-15	Negative strong [NH] [36] Negative [expected] [39]
Cognitive impairment	
Alzheimer Disease Related Quality of Life (ADRQL)	Positive [expected] [33, 35]
Well-being	
Cantril’s ladder	Positive strong [NH] [36] Positive [expected] [34, 39]
Environment and care quality	
Multiple deprivation scores of electoral ward	None [negative weak expected] [29]

Key: NH = No hypothesis stated

A complete table of summarised results regarding construct validity can be found in Supplementary Table 3.

The most commonly used sociodemographic comparator was age, and findings here were mixed. Of the studies using age as comparator, seven were conducted in Europe [16, 25, 27, 33, 35, 36, 39] (predominantly the UK) and all found a degree of negative association between increasing age and ICECAP-O score; of the three studies conducted in Australia [28, 40–42] two found no relationship and one [42] found a negative relationship. Findings were also mixed in relation to ‘living with others’: three European studies found positive relationships [17, 25, 27] and two studies conducted in Australia found no relationship [28, 42].

Other socio-demographic comparators used were gender [16, 27, 42], social class [27], employment status [28], income level [28, 36], receiving benefits, having a faith, being an unpaid carer [25] and being married [17]. The direction of the relationship between each of these comparators and ICECAP-O scores was found to be as hypothesised although those in relation to social class [28], employment status [28] and being an unpaid carer [27, 29] were not statistically significant.

The most commonly used comparator for general health was the EQ-5D which was used in ten studies [16, 27, 31, 33, 34, 36, 38–42, 44]. The relationship between EQ-5D and ICECAP-O scores was as expected in all studies, with one exception: Coast et al. [27] found no relationship between the EQ-5D score and Attachment (whereas the study by Keeley [16] found a positive relationship). Other generic health comparators used were: EQ-5D extended with a cognitive dimension (EQ-5D-3L + C) [33, 35], EQ-VAS [34, 36], SF-20 [39] and SF-36 [16]. A positive relationship between ICECAP-O scores and better health was both hypothesised and found within each of these studies.

Measures of physical health/independence that were used as comparators were: the Katz Index of Activities of Daily Living [39], the Barthel Activities of Daily Living index [33, 35, 36], Instrumental Activities of Daily Living, Physiological Profile Assessment, Short Physical Performance Battery [37, 38], the Care Dependency Scale [34], the Modified Rankin Scale [16], having a disability, pain or a limiting or long-term illness (a survey question), and doing moderate exercise [25]. All studies hypothesised and found a positive relationship between greater physical health/independence and ICECAP-O scores.

The following measures of (non-dementia) mental health were used as comparators: the Geriatric Depression Scale-15 [36, 39], the Hospital Anxiety and Depression Scale (HADS), the Herth Hope Index [40, 41]. All of the studies that stated a hypothesis expected a positive relationship between better mental health and overall ICECAP-O score. All but one study found a positive relationship, the exception being Makai et al. who found no relationship between the overall ICECAP-O score and the HADS.

Measures of cognitive impairment that were used as comparators were the Alzheimer’s Disease Related Quality of Life (ADRQL), the Mini–Mental State Examination and the Global Deterioration Scale [35]. All studies hypothesised and found a negative relationship between higher levels of cognitive impairment and ICECAP-O score.

The following measures of well-being were used as comparators: Cantril’s Ladder [34, 36, 39], Social Production Function Instrument for the Level of Well-being [39], Satisfaction With Life Scale (SWLS) and the Older People’s Quality of Life Questionnaire (OPQOL), and two survey questions about overall life satisfaction and narrative foreclosure in relation to the past and future [30]. All studies hypothesised and found a positive relationship between higher well-being and overall ICECAP-O index.

A range of other comparators were used that fall broadly under the heading of ‘environment and quality of care’. All studies hypothesised and found a positive relationship between ICECAP-O index scores and a ‘better’ environment (safer/less deprived/greater level of contact with others/better quality care).

#### Content validity

A summary of results relating to the assessment of content validity is presented in Table 2. Several studies found that participants questioned the relevance of the ICECAP-O domains. Studies found the domains were considered irrelevant for measuring outcomes in clinical trials of patients with hip fracture [18] or for carers of those with dementia trialling a Global Positioning Satellite technology [15]. Horwood et al. [19] found that in a UK population of surgical joint replacement patients, whilst some participants questioned the relevance of the Attachment and Security domains they were considering the relevance specifically in relation to aspects of their illness rather than in relation to their general quality of life. A ‘narrow’ interpretation of items considered not to be relevant was also observed in a study of older people in the Netherlands [21], again in relation to the ‘Attachment’ and ‘Security’ domains. However, in qualitative interviews with informal carers of people with dementia [20] in the UK, four themes were identified (social network and relationships; interactions with agencies; recognition of role; and time for oneself), which the researchers noted overlap with ICEAP-O domains. A Swedish study of over 70s found Attachment to be the most relevant domain and enjoyment the least [23].

**Table 2:** Content validity results

Paper	Findings	Population group
Haywood et al. (2014) [18]	Relevance: ICECAP domains considered to be not important or relevant	Clinical trial of patients with hip fracture (UK)
Horwood et al. (2014) [19]	Relevance: Some participants questioned why ‘love and friendship’ and ‘thinking about the future without concern’ were relevantHowever, they were focusing on the relevance to their operation	Patients undergoing joint replacement surgery (UK)
Van Leeuwen et al. (2015a) [21]	Relevance: Some participants narrowly interpreted ‘Attachment’ and ‘Security’ items respondents tending to concentrate on one aspect of a domain	Dutch older adults
Hörder et al. (2016) [23]	Relevance: Participants gave their highest relevance rating to Attachment and lowest to Enjoyment	Swedish 70-year-olds
Jones et al. 2014 [20]	Four themes were identified: social network and relationships; interactions with agencies; recognition of role; and time for oneself. All overlap with ICECAP-O domains	Carers of people with dementia (UK)
Milne et al. (2014) [15]	Several caregivers criticised the ICECAP-O for having questions that did not seem relevant	Carers of people with dementia (UK)

#### Responsiveness

A summary of the results relating to the assessment of responsiveness is presented in Table 3.

**Table 3:** Responsiveness results

Study	Anchor used to assess responsiveness	Findings
Keeley (2014) [16]	EQ-5D-3L EQ-5D-3L VAS Mini-Mental State Examination (MMSE) SF-36 sub-scale scores	Responsive in an RCT of blood pressure management for former stroke patients
Flynn et al. [17]	WOMAC score	Responsive among those undergoing total joint replacement (hip or knee)
Davis et al. (2017) [22]	Number of falls	Responsive among older adults with impaired mobility (fallen within last 12 months) particularly among those with mild cognitive impairment at baseline
Parsons et al. (2014) [24]	Oxford hip score	Not responsive amongst hip fracture patients undergoing surgery except at four weeks
Van Leeuwen et al. (2015b) [26]	Health global rating scale, Katz index of independence in activities of daily living, SF-12, quality of life global rating scale, Pearlin mastery scale, client-centred care questionnaire	Among frail older people receiving care mental health was most strongly associated with ICECAP-O over time

All but one study found the ICECAP-O to be responsive to change, the exception being a study based on hip fracture patients undergoing surgery [24]. In this study, the Oxford Hip Score was the anchor. However, the ICECAP-O was found to be responsive among those undergoing total joint replacement (hip or knee) surgery in a study that used the Western Ontario and McMaster Universities Arthritis Index (WOMAC) as an anchor.

#### Reliability

A summary of the results relating to the assessment of Reliability is presented in Table 4.

**Table 4:** Reliability results

Paper	Population	Analysis method
Hörder et al. (2016) [23]	Swedish 70 Year-Olds (non-patients)	Intraclass correlation coefficientKappa statistic
Van Leeuwen et al. (2015) [26]	Dutch frail older adults	Intraclass correlation coefficientStandard error of measurement

Only two studies assessed test–retest reliability [23, 26]. Both studies found good test–retest agreement with an Intraclass Correlation Coefficient (ICC) of greater than 0.7. Van Leeuwen et al. [26] found good agreement with a standard error of measurement of < 10% of the scale. However, Hörder et al. [23] used the Kappa Statistic and found systematic disagreement within each domain.

### Studies reporting data from the use of ICECAP in economic evaluations

#### Overview of the selected studies

Of the 22 studies in this category, twelve were undertaken in the UK, three in Australia [46–48], three in The Netherlands [49–52], two in Canada [53, 54], one in the USA [55] and one in Sweden [56].

Nine of the 22 studies reported results from full economic evaluations (assessing both costs and outcomes): six cost-effectiveness analyses [49, 50, 57–62] (two of these were feasibility studies), and three cost-consequence analyses [15, 63–65]. Thirteen studies reported partial economic evaluations, of which five were pilot or feasibility studies.

Some of the 22 studies evaluated interventions falling clearly within the health domain (such as telehealth [57, 58]; techniques/hardware used in hip fracture surgery [66, 67]; screening for those at risk of lung cancer [68]; comprehensive assessment and personalised clinical management strategies to reduce incontinence and nocturia for older adults following hip fracture [53]; comprehensive assessment for frail older people receiving acute hospital care [56]; and a pharmaceutical product [55]. However, most of the studies that were identified evaluated interventions relating to care in a community setting/self-care and/or integrated services for those with chronic and long-term conditions, where broader elements of quality of life (such as maintaining independence) would be more obviously and/or directly affected; the interventions here included: integrated health and social care [49, 52]; control over budgets for older people receiving community care in Australia [48]; a dementia self-management group [63]; a goal setting programme to promote healthy ageing and prevent dementia for those with low (or zero) needs [60]; information and communication technologies (ICT) training for those with a visual impairment [50]; interventions targeting post-acute needs [46, 54, 64, 65, 69]; and a programme of community activities aimed to help those with low (or zero) level needs to improve and maintain well-being [70]. Milne et al. studied the impact of GPS devices for those with a cognitive impairment [15] and Boots et al. assessed the impact of a blended care self-management program for family caregivers of people with early cognitive impairment [51].

All 22 studies used the ICECAP-O alongside other measures and in all but four studies [15, 51, 53, 56] this included the EQ-5D.

A full summary of study characteristics can be found in Supplementary Table 4.

### Methods for incorporating ICECAP-O data within economic evaluation

Twenty of the 22 studies used the ICECAP-O tariff values [8] to translate responses to the measure into an overall score; two studies [56, 68] were pilot/feasibility studies which aimed to test the feasibility of using the measure with particular patient populations, rather than analyse or interpret the results.

Eighteen of the 20 studies that generate an ICECAP-O tariff value also calculate either the change in ICECAP-O score induced by the treatment, or the component figures needed to calculate the change. One study [66] does not present results by arm due to the small sample size (this was a pilot/feasibility study) and one study [53] presents the correlation between presence of the condition and capability at baseline and follow-up. All but one of these 18 studies solely presents the results in tabular form alongside the corresponding results for other measures. The exception [67] summarises the results narratively.

Five of the 22 full and partial economic evaluation studies combine the change in ICECAP-O score with time [46, 49, 50, 52, 57, 58, 62]. Each study defines the results using different terminology: ‘An improvement from no capability to full capability on the ICECAP-O scale’ [57, 58]; ‘Capability QALY’ [49, 52]; ‘Years of well-being’ [50]; ‘QALY’ [46]; and ‘Capability Adjusted Life Years (CALYs)’ [62].

Three of the full economic evaluation studies undertook a net benefit analysis using the ICECAP-O and plotted a cost-effectiveness acceptability curve [49, 50, 52, 57, 58]. Two of the three studies also produced an ICER from the ICECAP-O results [50, 57, 58].

In general, relatively few papers with a focus on economic evaluation referred to ICECAP-O explicitly within the results and discussion sections (i.e., within the text as well as presenting results within a table or graph).

A full summary of the analysis and presentation of results can be found in Supplementary Table 5.

## Discussion

The number of publications assessing psychometric properties appears to have peaked in 2014, with a shift in emphasis now towards use of the measure within economic evaluation. It can reasonably be expected that interest in ICECAP-O, particularly since its inclusion within the NICE reference case for social care, will continue to increase.

Unsurprisingly, much of the research identified through this review was conducted within the UK (the country in which the measure was first developed and the only country, so far, within which a set of tariff values have been elicited from the population [8]), but research on and utilising the ICECAP-O has also been conducted across Europe and in English speaking countries such as Australia, Canada and the USA.

Generally, this review has identified evidence that ICECAP-O has good construct validity and responsiveness. Some papers have reported issues relating to content validity, but this issue appears to arise when respondents who have a clear medical need and have been recruited into a trial or other study because of having such a medical need, focus on that immediate medical need and consider broader aspects of quality of life (particularly relating to attachment and enjoyment) as being less relevant. Hence, this may largely be a contextual phenomenon.

Although there were promising findings from studies assessing psychometric properties from Europe, Canada and Australia, consideration should still be given as to whether attributes incorporated within ICECAP-O are culturally relevant for non-UK contexts as the measure is used in new settings, and whether the language is culturally appropriate even within non-UK English speaking contexts.

Reporting of results from ICECAP-O has, so far, been secondary to the reporting of results from health related measures such as EQ-5D (3L and 5L). Reporting of results for ICECAP-O has therefore tended to be brief, often with no discussion or interpretation of results. One factor that may be limiting the use and more rigorous reporting of results from ICECAP-O is the lack of guidance with respect to a decision-rule: whether (and how) capability well-being should be combined with time, and on what basis an intervention is judged to be cost-effective.

Five studies combined the ICECAP-O score with time, with each using different terminology. Whilst the phrases ‘An improvement from no capability to full capability’ and ‘Years of well-being’ are accurate and appropriate, these are just some of the variety of terms being used, and such varied terminology will be unhelpful in the long term. Mitchell et al. [71] and Goranitis et al. [72] have adopted the terms ‘years of full capability’ and ‘years of sufficient capability’ for ICECAP-A, and work is ongoing to identify a sufficient state of ICECAP-A and accompanying monetary threshold for a year of sufficient capability [73]. In line with these contributions, we would encourage use of the term ‘years of full capability’ by those using ICECAP-O in the short to medium term. What is clear, at present, is that terms such as ‘capability QALY’ are conceptually inaccurate and misleading, as the tariff values for ICECAP-O are not anchored on a scale from dead to full health.

Sufficiency represents an alternative normative approach to maximisation (as adopted in cost-utility analysis) and hence adoption of sufficiency would represent a further, significant, shift towards the ICECAP-O being used as a tool within a distinct conceptual framework. A significant programme of future research would be needed to identify a sufficient state of well-being, as defined by ICECAP-O. The issue of an appropriate monetary threshold would also potentially need to be addressed.

## Electronic supplementary material

Below is the link to the electronic supplementary material.

Supplementary material 1 (DOCX 42 KB)

## Footnotes

1 Those intending to use the ICECAP-O are requested to submit a User Agreement Form and information from that form is stored on a database.
