# Supplementary material for: ICECAP-O, the current state of play: a systematic review of studies reporting the psychometric properties and use of the instrument over the decade since its publication
Source: Qual Life Res. 2019 Jan 21;28(6):1429–39. doi: 10.1007/s11136-019-02114-y (PMC6522451; doi:10.1007/s11136-019-02114-y)
Supplement: Supplementary file 1 — Supplementary material 1 (DOCX 42 KB) [file 11136_2019_2114_MOESM1_ESM.docx]

# **ICECAP-O, the current state of play: A systematic review of studies reporting the psychometric properties and use of the instrument over the decade since its publication.**

***Quality of Life Research***

Louise Proud1, Carol McLoughlin1, Philip Kinghorn1*

1 Health Economics Unit, Institute of Applied Health Research, University of Birmingham, Edgbaston, B15 2TT, UK

* Corresponding author: p.kinghorn@bham.ac.uk

# **Supplementary Tables**

# **Supplementary Table 1: Data Extraction Form**

**Table 1A: Studies investigating psychometric properties**

| **Criteria** | **Justification** |
| --- | --- |
| **General study characteristics** | |
| Authors and publication year | Summary information for descriptive statistics |
| Property assessed | Each measurement property is a quality aspect of the instrument |
| Study design | Context of assessment |
| Condition | Instrument may perform differently in different populations and environments |
| Country |  |
| **Construct validity** | |
| Comparator | Instrument needs to be assessed against different comparators to generate evidence of the concepts it measures |
| Principle findings re evidence (measured by size of p-value ie statistical significance) of relationship between comparators and overall ICECAP-O score | To enable a narrative synthesis of results |
| Whether and to what extent this result is in line with the hypothesis | Is the result in line with the a-priori hypothesis |
| Principle findings re evidence (measured by size of p-value ie statistical significance) of relationship between comparator and individual ICECAP-O domains | As above |
| Whether and to what extent this result is in line with the hypothesis |  |
| **Content validity** | |
| A summary of principle findings re the relevance of the ICECAP-O domains | To enable a narrative synthesis of results |
| **Responsiveness** | |
| Anchor used to assess responsiveness | Instrument needs to be assessed using different anchors to generate evidence of its responsiveness to different concepts |
| Principle findings | To enable a narrative synthesis of results |
| **Reliability** | |
| Analysis method | Context of assessment |
| Principle findings | To enable a narrative synthesis of results |

**Table 1B: Studies reporting economic evaluation**

| **Criteria** | **Justification** |
| --- | --- |
| **General study characteristics** | |
| Authors and publication year | Summary information for descriptive statistics |
| Economic evaluation type |  |
| Condition | To understand the contexts within which the instrument is being used. Is it being used in those that are likely to lead to broader outcomes than health? |
| Intervention (or, for studies quantifying observational or survey data, the association the ICECAP-O was used to measure) |  |
| Country | Are the UK generated capabilities and values being used in non-UK settings? |
| ICECAP-O included alongside other measures (including EQ-5D (3L or 5L)) | Is the measure being used alongside the EQ 5D as per the NICE reference case? |
| **Analysis and presentation of ICECAP-O results** | |
| Algorithm used to translate ICECAP-O results into values? | To understand how the ICECAP-O results are being analysed, presented and interpreted. Are there any recurring gaps? Are results being interpreted in line with the conceptual basis of the instrument? How are the results being incorporated into economic evaluations given there is currently no agreed method for doing this. |
| Change in ICECAP-O score induced by treatment (or component figures) calculated? |  |
| Method of Presentation |  |
| Change in ICECAP-O score combined with time (If yes, how the result is defined/described/interpreted) |  |
| Other analysis/presentation of results in relation to economic evaluation |  |
| Were the results of the ICECAP-O discussed? |  |

# **Supplementary Table 2: General characteristics of studies investigating psychometric properties**

| **Authors Publication year** | **Property assessed** | **Study design** | **Condition** | **Country** |
| --- | --- | --- | --- | --- |
| Coast et al, 2008 | Validity (construct) | Quantitative: Cross-sectional | General population older people: UK | UK |
| Flynn et al, 2011 | Validity (construct) | Quantitative: Cross-sectional | General population older people: UK | UK |
| Flynn et al ca. 2011-14 | Validity (construct)  Responsiveness | Quantitative: Longitudinal | Joint surgery - hip or knee replacement surgery (pre and post surgery) | UK |
| Couzner et al, 2012; Ratcliffe et al, 2011 | Validity (construct) | Quantitative: Cross-sectional | Post-acute needs | Australia |
| Davis et al, 2013; Davis et al, 2012 | Validity (construct) | Quantitative: Cross-sectional | At risk of falling: recent (within 12 months) fall | Canada |
| Makai et al, 2012 | Validity (construct) | Quantitative: Cross-sectional | Cognitive impairment: moderate to high | The Netherlands |
| Makai et al, 2013 | Validity (construct) | Quantitative: Cross-sectional | Post-acute needs | The Netherlands |
| Couzner et al, 2013a | Validity (construct) | Quantitative: Cross-sectional | General population: Australia | Australia |
| Couzner et al, 2013b | Validity (construct) | Quantitative: Cross-sectional | General population older people: Australia Post-acute needs | Australia |
| Haywood et al, 2014 | Validity (content) | Semi Qualitative-Quantitative: Nominal Group Technique | Joint surgery - hip fracture surgery (post surgery) | UK |
| Horwood et al, 2014 | Validity (content) | Qualitative: 'Think aloud' study | Joint surgery - hip or knee replacement surgery (pre and post surgery) | UK |
| Jones et al, 2014 | Validity (content) | Qualitative: Semi-structured interviews | Informal carer (for someone with dementia) | UK |
| Keeley, 2014 | Validity (construct) Responsiveness | Quantitative: Cross-sectional and longitudinal | Previous stroke | UK |
| Makai et al, 2014 | Validity (construct) | Quantitative: Cross-sectional | Cognitive impairment: moderate to high | Germany |
| Van Leeuwen et al, 2015 a | Validity (content) | Qualitative: 'Think aloud' study | Frailty/social care needs | The Netherlands |
| Davis et al, 2016 | Validity (construct) | Quantitative: Cross-sectional | Cognitive impairment (mild) | Canada |
| Hackert et al, 2017 | Validity (construct) | Quantitative: Cross-sectional | Frailty/social care needs | UK |
| Sarabia-Cobo et al, 2017 | Validity (construct) | Quantitative: Cross-sectional | Cognitive impairment: moderate to high | Spain |
| Fortuin et al, 2018 | Validity (construct) | Quantitative: Cross-sectional | General population older people: The Netherlands | The Netherlands |
| Milte et al, 2018 | Validity (construct) | Quantitative: Cross-sectional | Joint surgery hip fracture surgery (post surgery) | Australia |
| Parsons et al, 2014 | Responsiveness | Quantitative: Longitudinal | Joint surgery - hip fracture surgery (pre and post surgery) | UK |
| Van Leeuwen et al, 2015 b | Reliability Responsiveness Validity (construct) | Quantitative: Cross-sectional and longitudinal and test retest | Frailty/social care needs | The Netherlands |
| Hörder et al, 2016 | Reliability Validity (content) | Quantitative: Test retest | General population : Sweden | Sweden |
| Davis et al, 2017 | Responsiveness | Quantitative: Longitudinal | At risk of falling: recent (within 12 months) fall without hip fracture | Canada |
| Milne et al, 2014 | Validity (content) | Quantitative: Cross-sectional | Cognitive impairment (mild, moderate and high) | UK |

# **Supplementary Table 3: Full list of summarised results regarding construct validity**

NH = No hypothesis stated, *only results significant at the 5% level were marked as significant (actual p-values were not reported). Therefore, results between 5% and 10% are not reported as significant (but would have been reported and classed as having ‘weak significance’ in other papers)

| **Comparator** | **Evidence (statistical significance) of relationship with overall capability [statements in square brackets indicate the degree to which the results were in line with the hypothesis]** | **Evidence (statistical significance) of relationship with capability dimensions [statements in square brackets indicate the degree to which the results were in line with the hypothesis]** |
| --- | --- | --- |
| **Socio-demographic characteristics** | | |
| Increased Age | None [expected] (Couzner et al, 2012; Ratcliffe et al, 2011)  Negative [NH] (Milte et al, 2018) ICECAP-O does not discriminate between over and under 65's [NH] (Couzner et al, 2013a) ICECAP-O discriminates between the young-old (65-75) and old-old (over 75) [expected] (Flynn et al, 2011, Hackert et al, 2017, Makai et al, 2013, Makai et al, 2014, Sarabia-Cobo et al, 2017) | Attachment: None [expected] (Coast et al, 2008) Security: None [expected] (Coast et al, 2008) Negative weak [none expected] (Keeley, 2014)  Role: Negative strong [negative weak expected] (Coast et al, 2008) Enjoyment: Negative weak [none expected] (Coast et al, 2008)  Control: Negative strong [negative weak expected] (Coast et al, 2008)  Negative weak [expected] (Keeley, 2014) |
| Living with others | None [positive expected] (Couzner et al, 2013a) Positive [expected] (Flynn et al, 2011)  None [NH] (Milte et al, 2018) | Attachment: positive [expected] (Flynn et al ca. 2011-14) |
| Living with marital partner | None [positive expected] (Couzner et al, 2013a) | Attachment: Positive strong [expected] (Coast et al, 2008)  Positive [expected] (Flynn et al ca. 2011-14)  Security: None [positive weak expected] (Coast et al, 2008)  Positive [expected] (Flynn et al ca. 2011-14)  Role: Positive weak [positive weak expected] (Coast et al, 2008)  Enjoyment: None [positive weak expected] (Coast et al, 2008)  Control: None [expected] (Coast et al, 2008) |
| Being married | NA | Attachment: positive [expected] (Flynn et al ca. 2011-14) |
| Gender (being female) | None [expected] (Keeley, 2014)  None [NH] (Milte et al, 2018) | Attachment: None [expected] (Coast et al, 2008)  Security: None [expected] (Coast et al, 2008)  Role: None [expected] (Coast et al, 2008)  Enjoyment: None [expected] (Coast et al, 2008)  Control: None [expected] (Coast et al, 2008) |
| Higher social class | None [positive expected] (Couzner et al, 2013a) | Attachment: None [expected] (Coast et al, 2008) Security: Weak positive [none expected] (Coast et al, 2008) Role: None [expected] (Coast et al, 2008) Enjoyment: None [expected] (Coast et al, 2008) Control: None [expected] (Coast et al, 2008) |
| Employment status | None to negligible [positive expected] (Couzner et al, 2013a) | NA |
| Income (Annual household income) | Positive when over $60k [positive expected] (Couzner et al, 2013a)  ICECAP-O discriminates between those on a higher income [NH] (Hackert et al, 2017) | NA |
| Receiving benefits | Negative [expected] (Flynn et al, 2011) | NA |
| Do not have a faith | Negative [expected] (Flynn et al, 2011) | NA |
| Provides informal care | None [negative expected] (Flynn et al, 2011) | Role: None [strong positive expected] (Coast et al, 2008) |
| **Generic health measures (index score)** | | |
| EQ-5D-3L or 5L | Positive strong [expected] (Hackert et al, 2017) Positive [expected] (Davis et al, 2013;Davis et al, 2012, Keeley, 2014) Positive [expected] (Makai et al, 2012) Positive [expected] (Makai et al, 2013)  Positive [expected] (Makai et al, 2014)  Positive [NH] (Milte et al, 2018)  ICECAP-O discriminates between:  above and below average health [NH] (Hackert et al, 2017) patients and the general population [expected] (Couzner et al, 2013b) | Attachment: None [expected] (Coast et al, 2008)  Positive [NH] (Keeley, 2014)  Security: Positive strong [positive weak expected] (Coast et al, 2008), Positive strong [NH] Keeley, 2014)  Role: Positive strong [expected] (Coast et al, 2008),  Positive [expected] (Couzner et al, 2012; Ratcliffe et al, 2011),  Positive [NH] (Keeley, 2014) Enjoyment: Positive strong [expected] (Coast et al, 2008)  None [positive strong expected] (Couzner et al, 2012; Ratcliffe et al, 2011) Positive strong [NH] (Keeley, 2014)  Control: Positive strong [expected] (Coast et al, 2008)  Positive weak [positive strong expected] (Couzner et al, 2012; Ratcliffe et al, 2011) Positive strong [NH] (Keeley, 2014) |
| EQ-5D-3L +C | Positive strong [expected] (Makai et al, 2014)  Positive strong [expected] (Sarabia-Cobo et al, 2017) | NA |
| EQ VAS | Positive strong [NH] (Makai et al, 2012)  Positive strong [NH] (Hackert et al, 2017) | NA |
| SF-20 | Positive strong [positive expected] (Makai et al, 2013) | NA |
| SF-36 | Positive [expected] (Keeley, 2014) | NA |
| **Physical health/independence** | | |
| Katz Index of Independence in Activities of Daily Living (ADL) | ICECAP-O discrimates between IADL dependent and non IADL dependent elderly [expected] (Makai et al, 2013) | NA |
| Barthel Activities of daily living (ADL) Index | Positive strong [expected] (Hackert et al, 2017, Makai et al, 2014) Positive strong [expected] (Sarabia-Cobo et al, 2017) | NA |
| Instrumental Activities of Daily Living | NA | Security: None*[NH] (Davis et al, 2013;Davis et al, 2012) Role: Negative [NH] (Davis et al, 2013;Davis et al, 2012) Control: Negative [NH] (Davis et al, 2013;Davis et al, 2012) |
| Care Dependency Scale (CDS) | Positive [expected] (Makai et al, 2012) ICECAP-O discriminates between higher and lower dependency [expected] (Makai et al, 2012) | NA |
| Modified Rankin Scale (MRS) | Negative [expected] (Keeley, 2014) | NA |
| Having a disability, pain or a limiting or long term illness (survey question) | Negative [expected] (Flynn et al, 2011) | Role:  Negative strong [expected] (Coast et al, 2008)  Negative strong [negative expected] (Flynn et al ca. 2011-14) Enjoyment:  Negative strong [expected] (Coast et al, 2008)  Negative strong [negative expected] (Flynn et al ca. 2011-14) Control:  Negative strong [expected] (Coast et al, 2008)  Negative strong [negative expected] (Flynn et al ca. 2011-14) |
| Physiological Profile Assessment (PPA) falls risk | None* [NH] (Davis et al, 2013;Davis et al, 2012) | Security: None* [NH] (Davis et al, 2013;Davis et al, 2012)  Role: None* [NH] (Davis et al, 2013;Davis et al, 2012)  Enjoyment: None* [NH] (Davis et al, 2013;Davis et al, 2012) Control: Negative [NH] (Davis et al, 2013;Davis et al, 2012) |
| Short Physical Performance Battery | None* [NH] (Davis et al, 2013;Davis et al, 2012) | Security: None* [NH] (Davis et al, 2013;Davis et al, 2012) Control: Negative [NH] (Davis et al, 2013;Davis et al, 2012) |
| Doing moderate exercise | None [positive strong expected] (Flynn et al, 2011) | NA |
| **Mental health (non-dementia)** | | |
| Geriatric Depression Scale-15 | Negative strong [NH] (Hackert et al, 2017) Negative [expected] (Makai et al, 2013) | NA |
| Hospital Anxiety and Depression Scale | None [NH] (Makai et al, 2012) | NA |
| Herth Hope Index | Positive [expected] (Couzner et al, 2012; Ratcliffe et al, 2011) | NA |
| **Cognitive impairment** | | |
| Global Deterioration Scale (GDS) | ICECAP-O discriminates between subgroups based on dementia severity (mild, moderate, and severe) [NH] (Sarabia-Cobo et al, 2017) | NA |
| Alzheimer Disease Related Quality of Life (ADRQL) | Positive [expected] (Makai et al, 2014, Sarabia-Cobo et al, 2017) | NA |
| Mini-Mental State Examination (MMSE) | ICECAP-O discriminates between severe and mild/moderate dementia [expected] (Makai et al, 2014) | Security: Positive [NH] (Davis et al, 2013;Davis et al, 2012) |
| **Wellbeing** | | |
| Older People's Quality of Life Questionnaire (OPQOL-13) | Positive strong [NH] (Hackert et al, 2017) | NA |
| Satisfaction With Life Scale (SWLS) | Positive strong [NH] (Hackert et al, 2017) | NA |
| Cantril’s Ladder | Positive strong [NH] (Hackert et al, 2017) Positive [expected] (Makai et al, 2012, Makai et al, 2013) | NA |
| Social Production Function Instrument for the Level of Well-being (SPF-IL) | Positive [expected] (Makai et al, 2013) | NA |
| Satisfaction with life (survey question) | Positive [expected] (Coast et al, 2008)  Positive [expected] (Makai et al, 2012) | NA |
| Narrative foreclosure re the past | Negative [expected] (Fortuin et al, 2018) | NA |
| Narrative foreclosure re the future | Negative [expected] (Fortuin et al, 2018) | NA |
| **Environment and care quality** | | |
| Multiple deprivation scores of electoral ward | None [negative weak expected] (Flynn et al, 2011) | Attachment: None [expected] (Flynn et al, 2011)  Security: None [expected] (Flynn et al, 2011) Role: None [expected] (Flynn et al, 2011)  Enjoyment: Negative [weak expected] (Flynn et al, 2011)  Control: Negative [weak expected] (Flynn et al, 2011) |
| Nature of locality and environment (survey question) | None [expected] (Coast et al, 2008) | NA |
| Feeling unsafe outdoors or indoors | Negative [expected] (Flynn et al, 2011) | NA |
| Contact with others (survey question) | NA | Attachment: Positive weak [strong expected] (Coast et al, 2008) Security: Positive strong [expected] (Flynn et al, ca.2011-14) Enjoyment: Positive [expected] (Coast et al, 2008) |
| 3-Item Care Transition Measure (CTM-3 ) | Positive [expected] (Couzner et al, 2012; Ratcliffe et al, 2011) | NA |

# **Supplementary Table 4: General characteristics studies reporting economic evaluation**

Key: CCA = Cost-consequence analysis, CEA = Cost-effectiveness analysis, CUA = Cost-utility analysis

| **Authors Publication year** | **Economic evaluation type** | **Condition** | **Intervention (or, for studies quantifying observational or survey data, the association the ICECAP-O was used to measure)** | **Country** | **ICECAP-O included alongside other measures (including EQ-5D (3L or 5L))** |
| --- | --- | --- | --- | --- | --- |
| Henderson et al, 2013; Hirani et al, 2014 | Full (CEA, CUA) | Chronic obstructive pulmonary disease, heart failure, diabetes | Telehealth | UK | Yes **(Including EQ-5D)** |
| Makai et al, 2015; Looman et al, 2016 | Full (CEA, CUA) | Frailty/social care needs | Integrated health and social care services | The Netherlands | Yes **(Including EQ-5D)** |
| Patty et al, 2018 | Full (CEA, CUA) | Visual impairment | Information and communication technologies (ICT) training | The Netherlands | Yes **(Including EQ-5D)** |
| Comans et al, 2013 | Partial  (effectiveness) | Post-acute needs | Post-acute rehab | Australia | Yes **(Including EQ-5D)** |
| Edmans et al, 2013 | Partial  (effectiveness) | Post-acute needs | Assessment, advice and referral | UK | Yes **(Including EQ-5D)** |
| Martinez et al, 2014 | Partial  (effectiveness) | Idiopathic pulmonary fibrosis | Drug | United States | Yes **(Including EQ-5D)** |
| Barker et al, 2017 | Partial  (effectiveness) | Neurological condition | Person-centred, community rehabilitation service | Australia | Yes **(Including EQ-5D)** |
| Bulamu et al, 2017 | Partial  (effectiveness) | Older people (aged 65 or over) receiving community aged care services | Community aged care services received under consumer directed care | Australia | Yes **(Including EQ-5D)** |
| Millard et al, 2017 | Partial  (effectiveness) | NA | Community activities | UK | Yes **(Including EQ-5D)** |
| Zusman et al, 2017 | Partial  (effectiveness) | Incontinence and nocturia | Post-acute information and advice | Canada | Yes **(EQ-5D NOT USED)** |
| Boots et al, 2018 | Partial  (effectiveness) | Family caregivers of people with early cognitive impairment (mild) | Blended care self-management program (electronic health support) | The Netherlands | Yes **(EQ-5D NOT USED)** |
| Milne et al, 2014 | Full (CCA) | Cognitive impairment (mild, moderate and high) | Global Positioning Device | UK | Yes **(EQ-5D NOT USED)** |
| Clare et al, 2015 | Full (CEA, CUA) | NA | Goal setting | UK | Yes **(Including EQ-5D)** |
| Bhattacharya et al, 2016 | Full (CEA, CUA) | On regular medication | Medication organisation devices | UK | Yes **(Including EQ-5D)** |
| Quinn et al, 2016 | Full (CCA) | Cognitive impairment early (mild) | Self-management skills | UK | Yes **(Including EQ-5D)** |
| Williams et al, 2016  Williams et al, 2017 | Full (CCA) | Post-acute needs | Post acute rehab enhanced | UK | Yes **(Including EQ-5D)** |
| Walters et al, 2017 | Full (CEA, CUA) | Mild frailty | Home-based health promotion | UK | Yes **(Including EQ-5D)** |
| Langford et al, 2015 | Partial  (effectiveness) | Post-acute needs | Information and advice | Canada | Yes **(Including EQ-5D)** |
| Griffin et al, 2016a | Partial  (effectiveness) | Hip fracture requiring surgery | Surgical technique/hardware | UK | Yes **(Including EQ-5D)** |
| Griffin et al, 2016b | Partial  (effectiveness) | Hip fracture requiring surgery | Surgical technique/hardware | UK | Yes **(Including EQ-5D)** |
| Neal et al, 2017 | Partial  (effectiveness) | At risk of lung cancer | Early x-ray | UK (England and Wales) | Yes **(Including EQ-5D)** |
| Westgard et al, 2018 | Partial  (effectiveness) | Frail older people receiving acute hospital care | Comprehensive geriatric assessment (CGA) | Sweden | Yes **(EQ-5D NOT USED)** |

# **Supplementary Table 5: Analysis and presentation of ICECAP-O results**

Tabular = ICECAP-O results presented alongside results from other measures in tabular form, QALY = Quality adjusted life year , ICER = Incremental cost effectiveness ratio, CEAC = Cost effectiveness acceptability curve

| **Authors Publication year** | **Economic evaluation type** | **Algorithm used to translate ICECAP-O results into values?** | **Change in ICECAP-O score induced by treatment (or component figures) calculated? (Method of Presentation)** | **Change in ICECAP-O score combined with time (If yes, how the result is defined/described/interpreted)** | **Other analysis/presentation of results in relation to economic evaluation** | **Were the results of the ICECAP-O discussed?** |
| --- | --- | --- | --- | --- | --- | --- |
| Henderson et al, 2013; Hirani et al, 2014 | Full (CEA, CUA) | Yes | Yes (Tabular) | Yes **(An improvement from no capability to full capability on the ICECAP-O scale)** | ICER CEAC | No |
| Makai et al, 2015; Looman et al, 2016 | Full (CEA, CUA) | Yes | Yes (Tabular) | Yes **(Capability QALY)** | CEAC | Yes |
| Patty et al, 2018 | Full (CEA, CUA) | Yes | Yes (Tabular) | Yes **(Years of wellbeing)** | ICER CEAC Cost effectiveness plane scatterplot Capability profile | Yes |
| Comans et al, 2013 | Partial  (effectiveness) | Yes | Yes (Tabular) | Yes **(QALYs)** | Capability profile | Yes |
| Edmans et al, 2013 | Partial  (effectiveness) | Yes | Yes - as an odds ratio; binary variables created by dichotomising the scores at the median level (Tabular) | No | None | Yes |
| Martinez et al, 2014 | Partial  (effectiveness) | Yes | Yes (Tabular) | No | None | Yes |
| Barker et al, 2017 | Partial  (effectiveness) | Yes | Yes (Tabular) | No | None | Yes |
| Bulamu et al, 2017 | Partial  (effectiveness) | Yes | Yes (Tabular) | No | Capability profile | Yes |
| Millard et al, 2017 | Partial  (effectiveness) | Yes | Yes (Forest plot of t-statistics - alongside those for other outcome measures and brief narrative summary) | No | None | No |
| Zusman et al, 2017 | Partial  (effectiveness) | Yes | No - as the correlation between the presence of UI and nocturia and capability at baseline & follow-up. (NA) | No | None | Yes |
| Boots et al, 2018 | Partial  (effectiveness) | Yes | Yes (Tabular) | No | None | Yes |
| Milne et al, 2014 | Full (CCA) | Yes | Yes (Tabular) | No | None | No (but feasibility of use discussed) |
| Clare et al, 2015 | Full (CEA, CUA) | Yes | Yes (Tabular) | No | None | No |
| Bhattacharya et al, 2016 | Full (CEA, CUA) | Yes | Yes (Tabular) | No | None | No (but feasibility of use discussed) |
| Quinn et al, 2016 | Full (CCA) | Yes | Yes (Tabular) | No | None | Yes |
| Williams et al, 2016  Williams et al, 2017 | Full (CCA) | Yes | Yes (Tabular) | No | None | Yes |
| Walters et al, 2017 | Full (CEA, CUA) | Yes | Yes (Tabular) | Yes **(Capability-adjusted life-years (CALYs))** | None | No (but feasibility of use discussed) |
| Langford et al, 2015 | Partial  (effectiveness) | Yes | Yes (Tabular) | No | None | No |
| Griffin et al, 2016a | Partial  (effectiveness) | Yes | Yes (Brief narrative summary) | No | None | No |
| Griffin et al, 2016b | Partial  (effectiveness) | Yes | No - results by arm not presented due to small sample size (NA) | NA | None | No |
| Neal et al, 2017 | Partial  (effectiveness) | No (ICECAP-O scores were not analysed at all - just the number and % of questionnaires completed) | No - ICECAP-O scores not analysed at all - just the number and % of questionnaires completed (NA) | NA | None | No (but feasibility of use discussed) |
| Westgard et al, 2018 | Partial  (effectiveness) | No (ICECAP-O scores were not analysed at all - study just looked at feasibility of using the questionnaire) | No - ICECAP-O scores not analysed at all - just the number and % of questionnaires completed (NA) | NA | None | No (but feasibility of use discussed) |
